# Antimicrobial activity of *Satureja Khuzestanica* Jamzad and *Satureja bachtiarica* Bunge essential oils against *Shigella flexneri* and *Escherichia coli* in table cream containing *Lactobacillus plantarum* LU5

**DOI:** 10.1002/fsn3.1871

**Published:** 2020-09-17

**Authors:** Seyed Mohammad Bagher Hashemi, Diako Khodaei

**Affiliations:** ^1^ Department of Food Science and Technology Faculty of Agriculture Fasa University Fasa Iran; ^2^ Department of Food Science and Technology Tarbiat Modares University Tehran Iran

**Keywords:** antibacterial assay, essential oils, gas chromatography, shelf life, table cream

## Abstract

The essential oils (EOs) from Marzeh khuzestani (*Satureja Khuzestanica* Jamzad) and Marzeh bakhtiari (*Satureja bachtiarica* Bunge) were analyzed and their antibacterial activities on *Shigella flexneri* and *Escherichia coli* in probiotic table cream containing *Lactobacillus plantarum* LU5 were evaluated. Carvacrol (86.5%) was the main component of Marzeh khuzestani, but thymol (33.5%), carvacrol (14.2%), borneol (13.4%), and linalool (11.5%) were the major constituents of Marzeh Bakhtiari EOs. Marzeh khuzestani exhibited the highest antibacterial/bactericidal activity on the tested bacteria. EOs combination showed no interaction on the *L. plantarum* but a synergism effect to inhibit the pathogen strains observed. Agar diffusion assay showed the highest inhibitory effect on *S. flexneri* (32.7 mm), *E. coli* (28.4 mm), and *L. plantarum* (24.7 mm) for the combination 2:1 Marzeh khuzestani:Marzeh Bakhtiari (*p* ≤ .05). The antibacterial activity of mixture of EOs in creams was evaluated and the sample contained of 1%k + 1%b showed the highest antibacterial activity after day 10 of storage (by lowering the number of *E. coli, S. flexneri*, and *L. plantarum* to 2.3, 1.9, and 1.4 log CFU/g compared to control sample). Overall acceptability of creams slightly decreased by the increase in EOs addition and the highest acceptability score of 7.9 observed for the sample contained 0.5%k + 0.5%b EOs. However, all treatments exhibited a high acceptance level that it confirms that the addition of EOs mixture had no effect on the sensorial attributes of the creams. The combination of tested EOs can be used as an antimicrobial agent in probiotic food products containing *L. plantarum*.

## Graphical abstract

Marzeh khuzestani and Marzeh bakhtiari EOs were added in probiotic table creams. Marzeh khuzestani EO exhibited the highest antibacterial/bactericidal activity. The combination of EOs had a synergism effect to inhibit pathogenic bacteria. Table creams containing different mixtures of EOs showed high total acceptability.

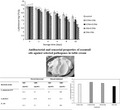

## 1 INTRODUCTION

Food preservation is an essential method to prevent the growth of spoilage or pathogen microorganisms such as bacterial and molds on the food. Nowadays and due to the health issues attributed to the chemical preservatives, there is a high tendency in food industry to replace the chemical preservatives with their natural alternatives (Hossain et al., 2016). Essential oils (EOs) are plants based and naturally occurring antimicrobials with a high potential biological activity. The antimicrobial effects of EOs in order to control the food spoilage have been reported in many researches (Hashemi, Niakousari, Saharkhiz, & Eskandari, 2011; Mohammadi & Aminifard, 2013; Olmedo, Nepote, & Grosso, 2013; Sumalan, Alexa, & Poiana, 2013; Tian et al., 2012). Recently, the demand for potential natural food preservatives with a broad spectrum of anti‐oxidant and antimicrobial activities to enhance the shelf life of perishable foods is increasing (Fratianni et al., 2010). Marzeh Khuzestani (*Satureja Khuzestanica* Jamzad) is an aromatic endemic plant growing wildly in the southern‐west side of Iran with a wide application as a traditional medicine due to its antiseptic and sedative properties (Hashemi, et al., 2012; Siavash Saei‐Dehkordi, Fallah, Heidari‐Nasirabadi, & Moradi, 2012). Marzeh Khuzestani EO is rich in natural monoterpenoid and carvacrol with diverse biological activities such as antimicrobial, antitumor, analgesic, anti‐inflammatory, anti‐parasitic, anti‐hepatotoxic, and hepatoprotective activities which makes it an excellent antimicrobial agent in food industry (Can Baser, 2008).

Marzeh Bakhtiari (*Satureja bachtiarica* Bunge) is another traditional medicine that is widely distributed in the central Zagros mountains, Iran. Antibacterial, antifungal, anti‐viral, anti‐oxidant, anti‐spasmodic, anti‐diarrheal, and anti‐inflammatory are some of the biological properties attributed to Marzeh Bakhtiari EOs. *P*‐cymene, carvacol, and thymol are the major components of the *S. bachtiarica* (Babadi, Ghasemi Pirbalouti, Nourafcan, & Hamedi, 2012).

In the term of food preservation, natural preservatives such as EOs are more suitable than chemical preservatives but the dosage and the costs for natural preservatives limit their application in food industry (Ju et al., 2019). Hence, evaluation of synergistic effect of combined EOs may be an effective way to solve this issue (Ju et al., 2020). Kong et al. (2016) reported a synergistic effect against *Fusarium solani* from combination thymol and salicylic acid which it was more than two‐fold higher than when it used alone. The synergistic effect of thymol and carvacol against *Pseudomonas aeruginosa* and *Staphylococcus aureus* has been reported by Lambert, Skandamis, Coote, and Nychas (2001). Ju et al. (2020) observed that the combination of eugenol and citral significantly improved the antifungal effects (3.4‐fold) against the main bread spoilage fungi (*Penicillium roqueforti* and *Aspergillus niger*) compared to each agent used separately.

Lactic acid bacterias (LABs) are one of the major groups of probiotics and they are widely used in the production of dairy products (Hashemi & Gholamhosseinpour, 2019). Some species of LABs, such as *Lactobacillus plantarum,* are commonly isolated from dairy products as well as fruits and vegetables and their role as probiotics in human health has been confirmed (Amin, Jorfi, Khosravi, Samarbafzadeh, & Farajzadeh Sheikh, 2009). In the case of the addition EOs into probiotic food, it is necessary that EOs exhibit a low inhibitory effect on the probiotic culture with an acceptable inhibitory activity on the pathogens. Table or coffee cream is an extremely viscous product contains 18% milk fat and can be fermented by the defined strains of LAB. This cream can be used as condiment along with the snacks or vegetables or also can be used as an ingredient in sauces or dressings (Tamime, 2009).

Therefore, the aim of the current research was to assess in vitro inhibitory potential of *Marzeh khuzestani* and *Marzeh bakhtiari* EOs alone or in combination against the growth of *Lactobacillus plantarum* LU5 as a probiotic culture or *Shigella flexneri*, and *Escherichia coli* as the pathogen bacteria and the effectiveness of these EOs as natural preservatives for table cream were evaluated.

## 2 MATERIALS AND METHODS

### 2.1 Bacterial strains

*L. plantarum* LU5 was obtained from Department of Food Science and Technology, Fasa University, Fasa, Iran. The probiotic potential of this strain was proved by Hashemi, Shahidi, Mortazavi, Milani, and Eshaghi (2014b). *L. plantarum* LU5 was reactivated in the MRS broth (Oxoid) at 37°C for 48 hr. *E. coli* PTCC 1399 and *S. flexneri* PTCC 1865 were purchased from Iranian Research Organization for Science and Technology, Tehran, Iran. The pathogenic bacteria were reactivated in nutrient broth (HiMedia) at 37°C for 24 hr.

### 2.2 Plant materials and essential oil extraction

Marzeh khuzestani (*Satureja khuzestanica*) and Marzeh bakhtiari (*Satureja bachtiarica* Bunge) with the moisture content of 13% (dry basis) were purchased from a local market in Kohgiluyeh and Boyer‐Ahmad province, Iran and kept at 4°C prior extraction. For essential oil extraction, hydrodistillation of 35 g of each dried plant in 500 ml distilled water was applied for about 3.5 hr by an all‐glass Clevenger‐type apparatus (British Pharmacopoeia, 1980).

### 2.3 Essential oil analysis

Gas Chromatography‐Mass equipment (GC‐MS; 6890N, Agilent Technologies) was used for determination of essential oils components. HP‐5MS capillary column (30‐m length; 0.25‐mm internal diameter; 0.25‐μm film thickness) was applied at a split ratio of 1:30, and the speed of helium was controlled at 1.3 ml/min. The oven temperature was adjusted at 60°C for 6 min, consequently enhanced to 280°C at 3°C/min (Hashemi, Niakousari, Saharkhiz, & Eskandari, 2014a; Hashemi et al., 2017).

### 2.4 Minimal inhibitory concentration (MIC) and Minimal bactericidal concentration (MBC)

Briefly, inoculums were prepared at 10^6^ CFU/ml and concentration range of each essential oil was adjusted at 0.8–100 µg/ml (dimethyl sulfoxide as solvent). Consequently, 95 µl of nutrient broth or MRS broth, 5 µl of each microbial inoculum, and 100 µl of each essential oil concentration were added to each well of the 96‐well plates. Afterward, incubation was performed at 37°C for 24 hr. For measurement of MBC, 100 μl of the microbial suspensions from MIC was cultured in nutrient agar and the lowest concentration with no growth was determined as MBC (Clemente, Aznar, & Nerín, 2019).

### 2.5 Fractional inhibitory concentration (FIC) and fractional bactericidal concentration (FBC)

Briefly, the different microorganism's suspensions in PBS, 20 μl of each EO concentration based on the MIC values and 1,760 μl of yeast extract were prepared. Afterward, the incubation was done at 37°C for 24 hr. The combination effect of both essential oils based on FIC value was reported as: antagonism, indifferent, additive, and synergy according to the method of Hyldgaard, Mygind, and Meyer (2012). For determination of the minimal bactericidal combination of essential oils, a method suggested by Mosquera, Sharp, Moore, Warn, and Denning (2002) was carried out to measure the FBC indicator. The following formula used for calculation of FIC and FBC;

$$\text{FIC or FBC}=\frac{\text{MIC or MBC of Marzeh khuzestani EO in combination}}{\text{MIC MBC of Marzeh khuzestani EO alone}}+\frac{\text{MIC or MBC of Marzeh bakhtiari EO in combination}}{\text{MIC or MBC of Marzeh bakhtiari EO alone}}$$

The antimicrobial interaction was calculated as FIC or FBC ≤ 0.5: synergic effect (where the combined antibacterial activity is greater than the sum of activity of both EOs when used separately); 0.5 < FIC or FBC ≤ 1: additive effect (where the combined antimicrobial activity is equal to sum of the activity of EOs acting jointly); 1 < FIC or FBC ≤ 4: no interactive effect; FIC or FBC > 4: antagonistic effect (where the combined antibacterial activity is lower than the sum of activity of both EOs used separately).

### 2.6 Combinatorial agar diffusion assays

Both essential oils were mixed at 25, 50, and 75% (v/v) concentration, and agar diffusion test was carried out for 100 μl of each bacterial suspension (10^6^ CFU/ml). Subsequently, 10 μl of each essential oil mixture was added to sterile filter disk (10 mm diameter) and placed on the nutrient agar or MRS + nutrient agar medium. After that, plates were incubated at 37°C for 24 hr (Hashemi et al., 2018).

### 2.7 Antibacterial activity of essential oils in table cream

The antibacterial activity of mixture of essential oils was measured on table cream. Table cream was purchased from Pegah Dairy Company and autoclaved for 15 min. After cooling, table cream was inoculated with 9.1–9.3 log CFU/g of each target strain separately and the mixture of essential oils (1 μl) was added to the samples. Samples were placed in polystyrene plastic tray covered by a lid and sealed with parafilm. Then, samples were kept at 4°C for 10 days and the count of each microorganism was detected during storage. For microbial determination, each sample was blended with peptone water in a stomacher (BagMixer 400 W, Interscience Co.) and following serial dilution; plating was carried out onto suitable medium. Afterward, plates were incubated at 37°C for 24–48 hr.

### 2.8 Sensory evaluation

Sensory assessment of table cream supplemented with essential oils was done by using a twelve‐member (six male and six female) semi‐trained panel. The panelists scored the overall acceptability aspects by using a 9‐point hedonic scale, where 1 = unacceptable and 9 = very acceptable, whereas the limit of acceptability was 6 (Hashemi, Amininezhad, Shirzadinezhad, Farahani, & Yousefabad, 2016).

### 2.9 Statistical analysis

Statistical analyses were performed with ANOVA and significant differences at *p* < .05 were carried out by Duncan's multiple range tests using SPSS package program (Version 22, SPSS Inc.).

## 3 RESULTS AND DISCUSSION

### 3.1 EOs composition

The composition of Marzeh khuzestani and Marzeh bakhtiari EOs is listed in Table 1. Carvacrol was the principle component of Marzeh khuzestani (86.5%), and the main components of Marzeh bakhtiari were thymol (33.5%), carvacrol (14.2%), borneol (13.4%), and linalool (11.5%). Thymol and carvacol are the most active oxygenated monoterpenes (Asensio, Grosso, & Juliani, 2015). Babadi et al. (2012) reported that the main constituent groups of *S. bachtiarica* EOs were monoterpenes and sesquiterpenes with the carvacrol (44.8%), gamma terpinen (18.7%), and thymol (14.95%) as the major components. Sefidkon and Jamzad (2000) also reported that thymol (44.5%), gamma terpinene (23.9%), *p*‐cymene (7.3%), β‐caryophyllene (5.3%), and borneol (4.2%) were the main components of Marzeh Bakhtiari EOs. Sefidkon, Jamzad, and Barazandeh (2005) reported that the EOs composition for *S. bachtiarica* was dependent on the region, so that for the Yazd population, the major components were carvacrol (66.5%), *p*‐cymene (15.2%) and linalool (4.6%) while for Fars population was carvacrol (49.3%), *p*‐cymene (12.7%), and trans‐α‐bergamotene (5.8%).

**TABLE 1:** Essential composition of Marzeh khuzestani and Marzeh bakhtiari

Compound	RI	Marzeh khuzestani	Marzeh bakhtiari
α‐Pinene	939	0.4 ± 0.1	0.5 ± 0
Camphene	948	—	0.6 ± 0
β‐Pinene	978	0.4 ± 0.1	0.3 ± 0.1
Myrcene	983	1.1 ± 0.1	—
p‐Cymene	1,018	0.5 ± 0	3.1 ± 0.1
Limonene	1,026	0.8 ± 0.2	—
γ‐Terpinene	1,063	1 ± 0.1	—
trans‐Sabinene hydrate	1,075	0.4 ± 0	0.8 ± 1
Linalool	1,099	0.5 ± 0.1	11.5 ± 0.3
Camphor	1,123	—	1.6 ± 0.2
Borneol	1,163	0.6 ± 0.1	13.4 ± 0.3
Terpine‐4‐ol	1,164	0.7 ± 0.2	2.1 ± 0.1
Carvacrol methyl ether	1,242	0.4 ± 0	—
Thymol	1,292	1.4 ± 0.2	33.5 ± 0.4
Carvacrol	1,302	86.5 ± 0.5	14.1 ± 0.3
Carvacryl acetate	1,348	—	5.1 ± 0.2
β‐Caryophyllene	1,432	0.5 ± 0	0.9 ± 0.1
β‐Bisabolene	1,528	0.8 ± 0.1	—
Caryophyllene oxide	1,586	—	12.3 ± 0.2

Note

Values are means ± standard error.

Hadian, Hossein Mirjalili, Reza Kanani, Salehnia, and Ganjipoor (2011) reported that carvacrol was the major component of the eight populations of *S. khuzistanica*. Farsam, Amanlou, Radpour, Salehinia, and Shafiee (2004) also reported that carvacrol was the main component in *S*. *khuzistanica* (93.9%) followed by eugenol (1.0%), and *p*‐cymene (0.8%). Carvacrol is a monoterpenoid phenol, and it is one of the major component of EOs from *Lamiaceae* family (such as thyme, oregano, and savory oil) (Hadian et al., 2011). A wide range of activities such as antimicrobial, anti‐oxidant, anti‐candidal, and anti‐inflammatory characterization are attributed to the carvacrol (Di Pasqua et al., 2007). Other researchers have reported carvacrol as the major compound in *S. khuzistanica* EOs (Hashemi et al., 2012; Mazarei & Rafati, 2019). The hydrophobicity of EO components such as carvacrol due to their lipophilic nature possesses a high affinity for cell membranes and causes physicochemical changes in microorganisms’ membrane that cause the decrease in membrane integrity and potential depolarization (Mazarei & Rafati, 2019).

### 3.2 Minimal inhibitory concentration (MIC) and Minimal bactericidal concentration (MBC) of EOs

The antimicrobial activity of Marzeh khuzestani and Marzeh bakhtiari EOs was determined against *L. plantarum* as a common food probiotic strain and against *S. flexneri* and *E. coli* as food‐borne bacteria. The MIC and MBC obtained are presented in Table 2. The results showed that both EOs were effective against tested bacteria. However, the difference in MIC and MBC of EOs observed. Marzeh Khuzestani EO with MIC ranging from 3.125 to 12.5 (µg/ml) exhibited a higher antibacterial property. However, the MIC for Marzeh bakhtiari EO was ranged from 6.25 to 25 (µg/ml). EOs containing aldehydes or phenols such as cinnamaldehyde, citral, carvacrol, eugenol, or thymol as the major components have been reported to have the highest antibacterial activity, followed by EOs containing terpene alcohols (Bassolé & Juliani, 2012). *S. flexneri* was the most sensitive pathogen strain to both EOs but *L. plantarum* and *E. coli* had a similar sensitivity to the EOs. These results are promising in term of production probiotic food and EOs have less inhibitory effect on the LABs that are the main probiotic culture in dairy products. Pirbalouti et al. (2010) observed a higher antimicrobial activity against gram positive bacteria and yeasts than gram negative bacteria for *S. bachtiarica* EOs and they reported that *Bacillus cereus* and *Candida albicans* were most sensitive strains against the EO. Babadi et al. (2012) reported that carvacrol and thymol are the major bactericidal components of *S. bachtiarica* EO and result in disturbance in bacteria membrane layer and leakage of intercellular ATP and potassium ions that cause cells death.

**TABLE 2:** Minimum inhibitory concentration (MIC) and minimum bactericidal concentration (MBC) of Marzeh khuzestani and Marzeh bakhtiari against tested bacteria

Bacterial strains	Marzeh khuzestani		Marzeh bakhtiari	
	MIC (µg/ml)	MBC (µg/ml)	MIC (µg/ml)	MBC (µg/ml)
*L. plantarum* LU5	12.5	25	25	100
*S. flexneri*	3.125	6.25	6.25	12.5
*E. coli*	12.5	25	25	100

Although a higher concentration of EOs required to exhibit bactericidal activity (MBC) than MIC, a similar pattern observed for the MBC of the EOs and it ranged from 6.25–25 and 12.5–100 for the Marzeh khuzestani and Marzeh bakhtiari, respectively. A higher bactericidal activity observed for the Marzeh khuzestani EO and *S. flexneri* was the most sensitive strain.

### 3.3 Fractional inhibitory concentration (FIC) and fractional bactericidal concentration (FBC)

The combination effect of the Marzeh khuzestani and Marzeh bakhtiari EOs evaluated, and the fractional inhibitory effects (FIC) and fractional bactericidal concentration (FBC) against the selected bacteria are presented in Table 3. The values of FIC and FBC were uniformly coincident and the results showed synergic effect of the EOs combination against both *S. flexneri* and *E. coli* with the FIC and FBC values of 0.5 but additive effect against *L. plantarum* strain observed (FIC and FBC of 0.75). It can be concluded that the combination of both EOs had no synergistic effect on the probiotics while it significantly prohibited the pathogen bacteria and it is promising in term of application the EOs mixture in probiotic food products. These observations affirm the potential use of combination of Marzeh khuzestani and Marzeh bakhtiari EOs on food pathogen bacteria.

**TABLE 3:** Fractional inhibitory concentration (FIC) and fractional bactericidal concentration (FBC) indices of Marzeh khuzestani and Marzeh bakhtiari against tested bacteria

Bacteria	FIC	FBC
*L. plantarum* LU5	0.75	0.75
*S. flexneri*	0.5	0.5
*E. coli*	0.5	0.5

FIC ≤ 0.5: synergic effect; 0.5 < FIC≤1: additive effect; 1 < FIC≤4: no interactive effect; FIC > 4: antagonistic effect.

Synergistic effects between carvacrol and other hydrocarbon monoterpense (e.g., α‐pinene, camphene, myrcene, α‐terpinene, and *p*‐cymene) have been reported previously by other researchers that hydrocarbons by interacting with bacterial cell membrane facilitate the carvacrol penetration into the cells (De Azeredo et al., 2011; Ultee, Slump, Steging, & Smid, 2000). Minor components by modulating the activity of the major components help the synergism of EOs. Moon and Rhee (2016) reported a synergistic effect between carvacrol and thymol against *E. coli*, *S*. Typhimurium and *L. monocytogenes*.

### 3.4 Combinatorial agar diffusion assays

Three different combinations of Marzeh khuzestani:Marzeh bakhtiari (1:1, 1:2, and 2:1) were prepared, and their antimicrobial inhibition zone against selected bacteria is presented in Table 4. As can be seen, the combination of 2:1 Marzeh khuzestani:Marzeh bakhtiari exhibited the strongest antibacterial activity compared to the other treatments (*p* ≤ .05) and reduced the growth of bacteria strains with the inhibition zones from 24.7 mm for *L. plantarum* to 32.7 mm for *S. flexneri*. However, the combinations of 1:2 and 1:1 of Marzeh khuzestani:Marzeh Bakhtiari exhibited the similar antibacterial activities against the selected bacteria (*p* ˃ .05). This observation affirms a higher antimicrobial activity of Marzeh khuzestani compared to Marzeh bakhtiari EOs that it was also confirmed in MIC and MBC tests. The comparison of the sensitivity of microorganism to the antimicrobial properties of different combinations of EO showed that *S. flexneri* was the most sensitive strain (*p* ≤ .05), followed by *E. coli*, and *L. plantarum*. The amount of carvacrol in Marzeh khuzestani EO is ~6 times higher than that of Marzeh Bakhtiari EO. The high antimicrobial properties of carvacrol against *S. flexneri* have been reported by Bagamboula, Uyttendaele, and Debevere (2004).

**TABLE 4:** Antibacterial activity of Marzeh khuzestani and Marzeh bakhtiari against tested bacteria

	Marzeh khuzestani + Marzeh bakhtiari (1:2 (v/v))	Marzeh khuzestani + Marzeh bakhtiari (1:1 (v/v))	Marzeh khuzestani + Marzeh bakhtiari (2:1 (v/v))
Bacterial strains	Inhibition zone (mm)	Inhibition zone (mm)	Inhibition zone (mm)
*L. plantarum* LU5	21.5 ± 0.4^bB^	22.8 ± 0.5^bB^	24.7 ± 0.8^cA^
*S. flexneri*	28.4 ± 0.5^bB^	29.6 ± 0.3^aB^	32. 7 ± 0.3^aA^
*E. coli*	22.1 ± 0.3^bB^	23.3 ± 0.5^bB^	28.4 ± 0.7^bA^

Note

Values are means ± standard error. Within each column means with the same lowercase letters are not significantly different (*p* > .05). The same uppercase letters are not significantly different between different concentrations for each method used (*p* > .05).

### 3.5 Antibacterial activity of essential oils in table cream

**FIGURE 1:**
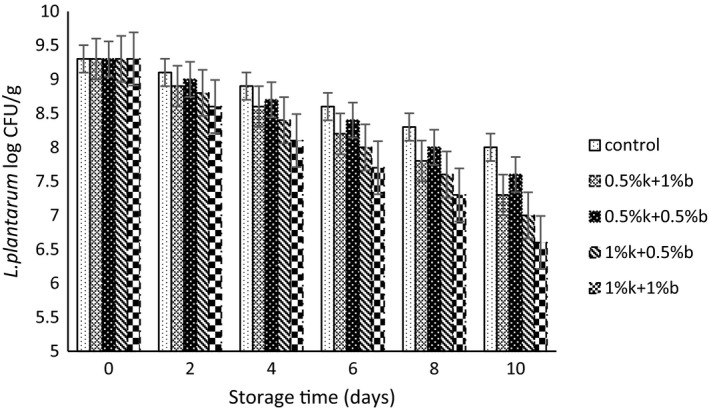
The population change for *L. plantarum* in table creams treated with different concentrations of Marzeh khuzestani (k) and Marzeh bakhtiari (b) during 10 days of storage

**FIGURE 2:**
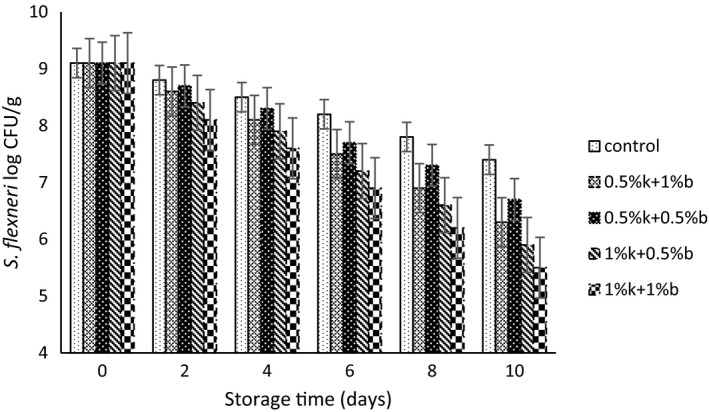
The population change for *S. flexneri* in table creams treated with different concentrations of Marzeh khuzestani (k) and Marzeh bakhtiari (b) during 10 days of storage

**FIGURE 3:**
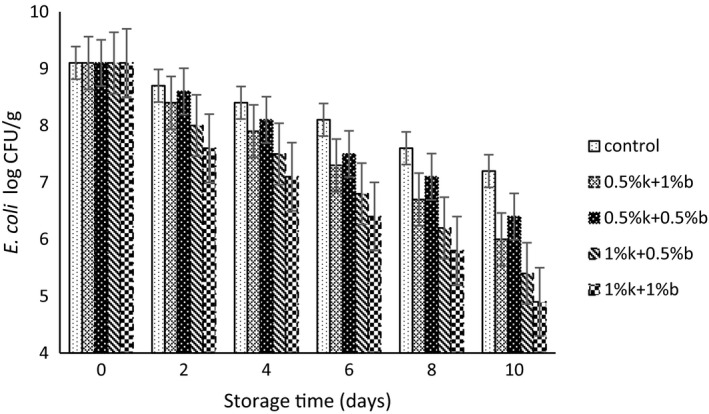
The population change for *E. coli* in table creams treated with different concentrations of Marzeh khuzestani (k) and Marzeh bakhtiari (b) during 10 days of storage

The population changes for *L. plantarum*, *S. flexneri*, and *E. coli* strains in table creams containing different combinations of EOs during storage period have been depicted in Figures 1, 2, 3, respectively. The initially recorded population for *L. plantarum* in control samples was 9.3 log CFU/g and a reduction of 1.3 log CFU/g observed after 10 of storage. However, the addition of EOs significantly reduced the initial number of viable *L. plantarum* cells in compared to control samples and the treatments containing higher concentration of EOs exhibited a lower viable cells of *L. plantarum*. Treatments containing 1%k + 1%b EOs and 1%k + 0.5%b showed the lowest population during the storage up to the day 8 of storage. The lowest population of probiotic strain observed at the day 10 of storage and for the 1%k + 1%b treatment that contained the highest amount of EOs. Moritz, Rall, Saeki, and Fernandes Júnior (2012) reported that the population for *L. rhamnosus* in fermented milk did not affect by the addition of clove and mint EOs.

Similar pattern observed for *E. coli* and *S. flexneri* population during the storage of table cream samples. The population of both bacteria strain decreased during the storage time, and for the treatments with higher amount of EOs addition (1%k + 0.5%b and 1%k + 1%b), the number of viable bacteria was significantly lower than the other treatments up to the day 6 of storage. However, the difference between treatments containing 0.5%k + 0.5%b and 0.5%k + 1%b EOs was not significant (*p* > .05). Treatment loaded with 1%k + 1%b EOs exhibited the lowest number of pathogen strains during the storage period so that, it decreased the viable number of *S. flexneri* and *E. coli* cells up to 1.9 and 2.3 log CFU/g by the end of storage time. Govaris, Botsoglou, Sergelidis, and Chatzopoulou (2011) reported that oregano and thymol EOs showed strong antimicrobial activity against the *L. monocytogenes* and *E. coli* in feta cheese.

### 3.6 Sensory analysis

**FIGURE 4:**
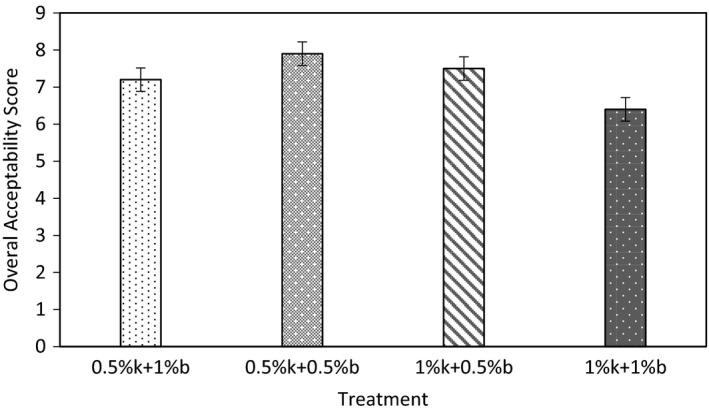
Overall acceptance score for the table creams incorporated with different amount of Marzeh khuzestani (k) and Marzeh bakhtiari (b)

Sensory evaluation scores of the creams treated with different combination ratios of Marzeh khuzestani and Marzeh bakhtiari during refrigerated storage at 4°C are shown in Figure 4. Results showed that by increase in the EOs addition in the cream, a slight decrease in overall acceptability was observed. So that, the lowest acceptability of 6.4 observed for the sample containing 1%k + 1%b EOs and the highest acceptability were in sample contained 0.5%k + 0.5%b EOs. However, all treatments exhibited a high acceptance level (higher than the limit of acceptability of 6) that it confirms that addition of EOs mixture had no negative effect on the sensorial attributes of the table creams. Moon and Rhee (2016) reported that addition of carvacrol and thymol EOs in soy sauce up to 1.0 mM had no significant effect on the sensorial attributes. The use of EOs in food and as preservative is limited since they can cause unpleasant smells/flavors when used at effective doses (Angienda & Hill, 2011). Therefore, using a combination of EOs due to their synergism effect leads into lower effective doses. Clemente et al. (2019) also reported that the combination of mustard and cinnamon EOs had no negative effect on the organoleptic properties of Spanish bread.

## 4 CONCLUSION

Marzeh khuzestani and Marzeh bakhtiari EOs were taken to evaluate their antibacterial efficiency alone or in combination against *L. plantarum*, *S. flexneri*, and *E. coli*. GC‐MS analysis showed that the major compounds in Marzeh khuzestani EO were carvacrol, while thymol, carvacrol, borneol, and linalool were the major compounds in Marzeh bakhtari EO. MIC and MBC analysis showed higher antibacterial activity in Marzeh khuzestani EOs. The combination of EOs exhibited a synergistic effect against the pathogen bacteria, while an additional effect observed for *L. plantarum*. The mixture of 2:1 khuzestani:Marzeh bakhtiari showed the highest inhibitory effect on the selected bacteria and in Agar diffusion test. The shelf life of table creams containing different mixtures of the EOs and inoculated with the selected bacteria was evaluated and it was observed that the highest antibacterial activity against the pathogens was at the treatment with EOs combination of 1%k + 0.5%b and 1%k + 1%b but *L. plantarum* showed the lowest dependency on the EOs. The total acceptability test for the treatments with different amounts of EOs showed that all treatments showed a high acceptance level that confirms that the addition of Marzeh khuzestani and Marzeh bakhtiari at a high level had no negative effect on sensory acceptance of the table creams and it can significantly improve the microbiological properties of the product.

## CONFLICT OF INTEREST

The authors declare that they have no conflict of interest.

## ETHICAL APPROVAL

This study does not involve any human or animal testing.

## INFORMED CONSENT

Written informed consent was obtained from all study participants.
